# Allogeneic and other innovative chimeric antigen receptor platforms

**DOI:** 10.46989/001c.121404

**Published:** 2024-09-27

**Authors:** Andrew P Jallouk, Salyka Sengsayadeth, Bipin N Savani, Bhagirathbhai Dholaria, Olalekan Oluwole

**Affiliations:** 1 Medicine, Hematology Oncology Vanderbilt University Medical Center

**Keywords:** Chimeric antigen receptor, Allogeneic, Engineered cellular therapy, Natural killer cell, T cell, Macrophage

## Introduction

Chimeric antigen receptor (CAR) T-cell therapy has revolutionized the treatment of hematologic malignancies. Since the initial approval of tisagenlecleucel and axicabtagene ciloleucel in 2017, the number of CAR T-cell infusions performed annually has grown exponentially, and the number of indications continues to expand. As of December 2023, six CAR T-cell products have been approved by the United States Food and Drug Administration (FDA), four for a variety of lymphoid malignancies (diffuse large B-cell lymphoma, follicular lymphoma, acute lymphoblastic leukemia, etc.) and two for multiple myeloma (MM). Despite their impressive efficacy, notable challenges exist which continue to limit patient access to these life-saving therapies.^1,2^ In particular, their cost and logistical burdens, including the supportive care necessary to safely deliver them, have restricted their use to large referral centers and made adoption by community practitioners difficult.

All of the CAR T-cell products that are currently FDA-approved are autologous in nature, meaning that each dose must be individually manufactured for each patient using the patient’s own cells. This process occurs in a centralized manufacturing facility and typically requires several weeks from T-cell collection to re-infusion. This lengthy manufacturing time limits the use of these therapies for aggressive malignancies, and frequently requires the use of bridging therapies to prevent symptomatic disease progression or even death.^3^ Furthermore, manufacturing bottlenecks with certain CAR T-cell products have substantially limited the number of patients who can receive them, and the possibility that a manufactured product will not meet technical specifications introduces further uncertainty regarding whether a patient planned for CAR T-cell therapy will ultimately receive their infusion.^4^

In addition to these logistical challenges, the autologous nature of the current manufacturing process results in heterogeneous products, whose variable quality may negatively impact efficacy and toxicity. CAR T-cell phenotype is felt to be a key determinant of efficacy, and several studies have examined in detail the association between infusion product characteristics and clinical outcomes.^5,6^ Concerns regarding the impact of prior cancer-directed therapies on T-cell fitness have provided motivation for avoiding treatments such as bendamustine, if CAR T-cell therapy is planned,^7^ and attempting to incorporate CAR T-cells into earlier lines of therapy.^8^ Toxicity is another important limitation of the currently available CAR T-cell products. While the rates of cytokine release syndrome (CRS) and immune effector cell-associated neurotoxicity syndrome (ICANS) vary by product, they remain high enough so that CAR T-cell therapy is generally available only at experienced centers, capable of providing the appropriate supportive care and implementing the FDA-mandated risk evaluation and mitigation strategies (REMS) programs. Several strategies, including the use of prophylactic corticosteroids^9^ and prophylactic anakinra,^10,11^ have been proposed to reduce treatment-related toxicity. However, the impact on toxicity incidence and severity has been relatively modest, and alternative platforms which fully eliminate these toxicities would be of great benefit.

Recently, the development of bispecific antibodies targeting both CD3 on recipient T-cells and tumor antigens such as CD19 and CD20 has provided a potential alternative to autologous CAR T therapy.^12,13^ Bispecific antibodies are logistically simpler to deliver as they are immediately available and typically associated with lower-grade toxicities than CAR T-cell therapy. Nevertheless, they too depend on the fitness of the recipient’s immune system to exert their effect and, unlike CAR T-cells, require extended or indefinite dosing regimens. Furthermore, although bispecific antibodies and CAR T-cells have not been directly compared, the observed response rates with bispecific antibodies are typically lower than those seen with CAR T-cells in similar lines of therapy.^14–17^ Thus, there remains a pressing need for advancements to enhance the efficacy and accessibility of the CAR T platform.

This review will cover innovative CAR platforms which seek to overcome the limitations of currently available therapies and facilitate continued development of this exciting technology.

## Allogeneic CAR T-cell Therapy

**Figure 1.**
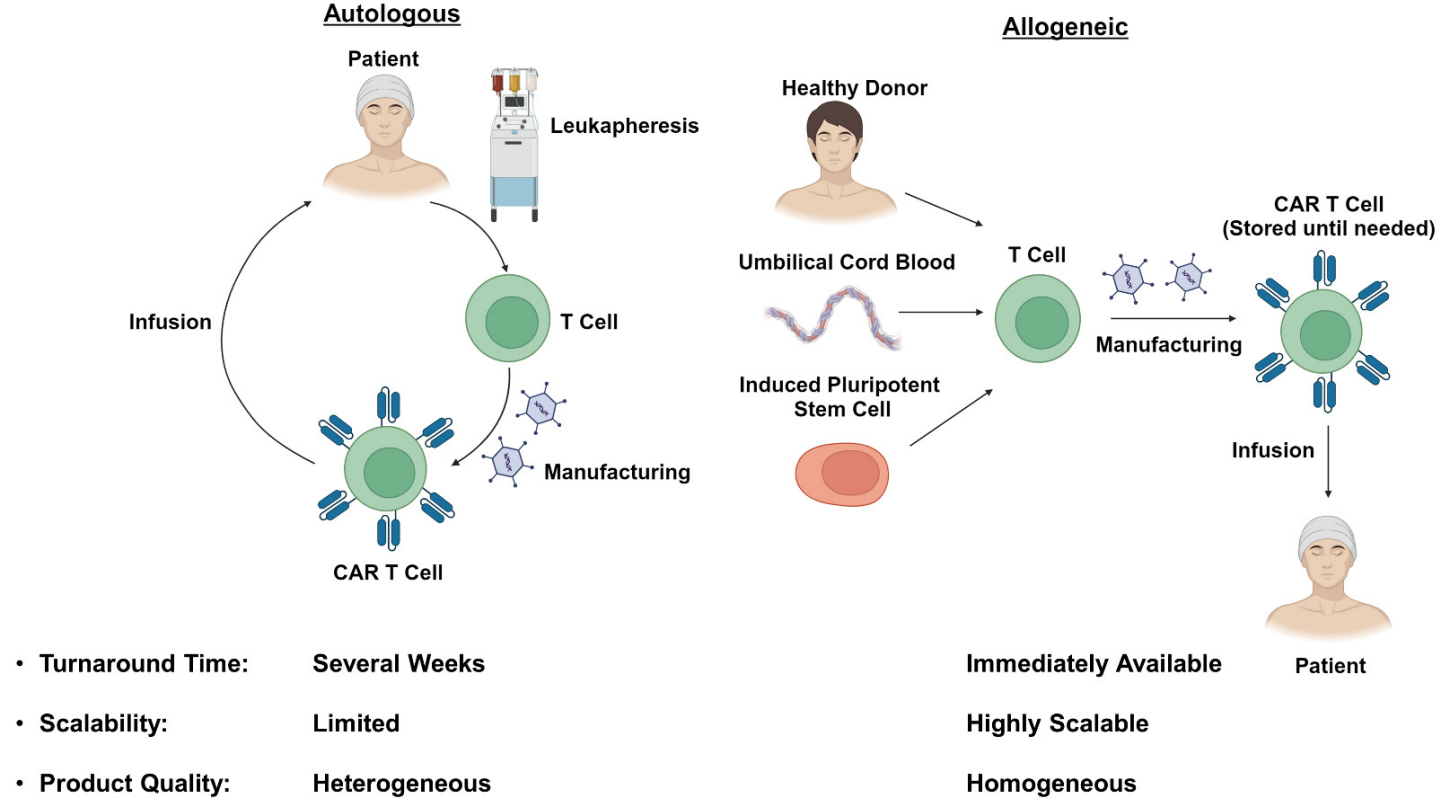
Comparison of autologous and allogeneic CAR T-cell therapies

As many of the limitations of autologous CAR T-cell therapies are related to the need for individual dose manufacturing, there has been substantial interest in developing allogeneic “off-the-shelf” therapies (**Figure 1**). These treatments would be immediately available when needed, and would reduce the cost by taking advantage of economies of scale during the manufacturing process.^18,19^ Additionally, the use of healthy donors without prior exposure to chemotherapy may improve T-cell fitness and the efficacy of the resulting CAR T product. Nevertheless, the use of allogeneic cells has its own unique challenges which require innovative strategies to overcome.

The currently available autologous CAR T-cell therapies are each manufactured from a single leukapheresis product collected from the patient of interest. While this strategy provides sufficient material to generate multiple CAR T doses, the desire to produce hundreds or even thousands of doses from a single donor has prompted exploration of alternative cell sources.^18,20^ Umbilical cord blood has been proposed as a source of antigen-naïve T-cells with enhanced tumor-reactive characteristics.^21–23^ Induced pluripotent stem cells (iPSCs) have also been proposed as a source of allogeneic CAR T-cells, and would be particularly attractive as they, theoretically, have unlimited capacity to self-renew and could be used indefinitely.^24–26^ Regardless of the cell source, the phenotype of the resulting T cells is of paramount concern. Several studies have identified a stem cell memory T (T_SCM_) phenotype which is long-lived, self-renewing and capable of giving rise to effector populations responsible for antitumor activity.^27–29^ This cell type would serve as an ideal component of an allogeneic CAR T-cell product.^30,31^ In contrast, exhausted CD8^+^ T cell populations in autologous CAR T infusion products have been associated with poor clinical responses and would preferably be avoided.^5,32^ Refinement of the allogeneic CAR T-cell manufacturing process to optimize T-cell phenotype while maintaining scalability will be an important challenge moving forward.

One of the early concerns with allogeneic CAR T-cell therapy was the potential for graft-versus-host disease (GVHD) caused by recognition of recipient antigens by the endogenous allogeneic αβ T-cell receptor (TCR). This concern has been effectively ameliorated through genetic knockout of the TCR followed by depletion of any residual cells expressing an αβ TCR.^33^ In fact, no cases of GVHD were reported in any of several phase-1 trials conducted using a variety of different allogeneic CAR T-cell products, all of which had genetic knockout of the *TRAC* locus to prevent TCR expression.^34–38^ Other strategies to avoid GVHD include the use of T-cells expressing a virus-specific TCR^39,40^ or the use of alternative cell types which do not cause GVHD, as will be described on subsequent sections.

**Figure 2.**
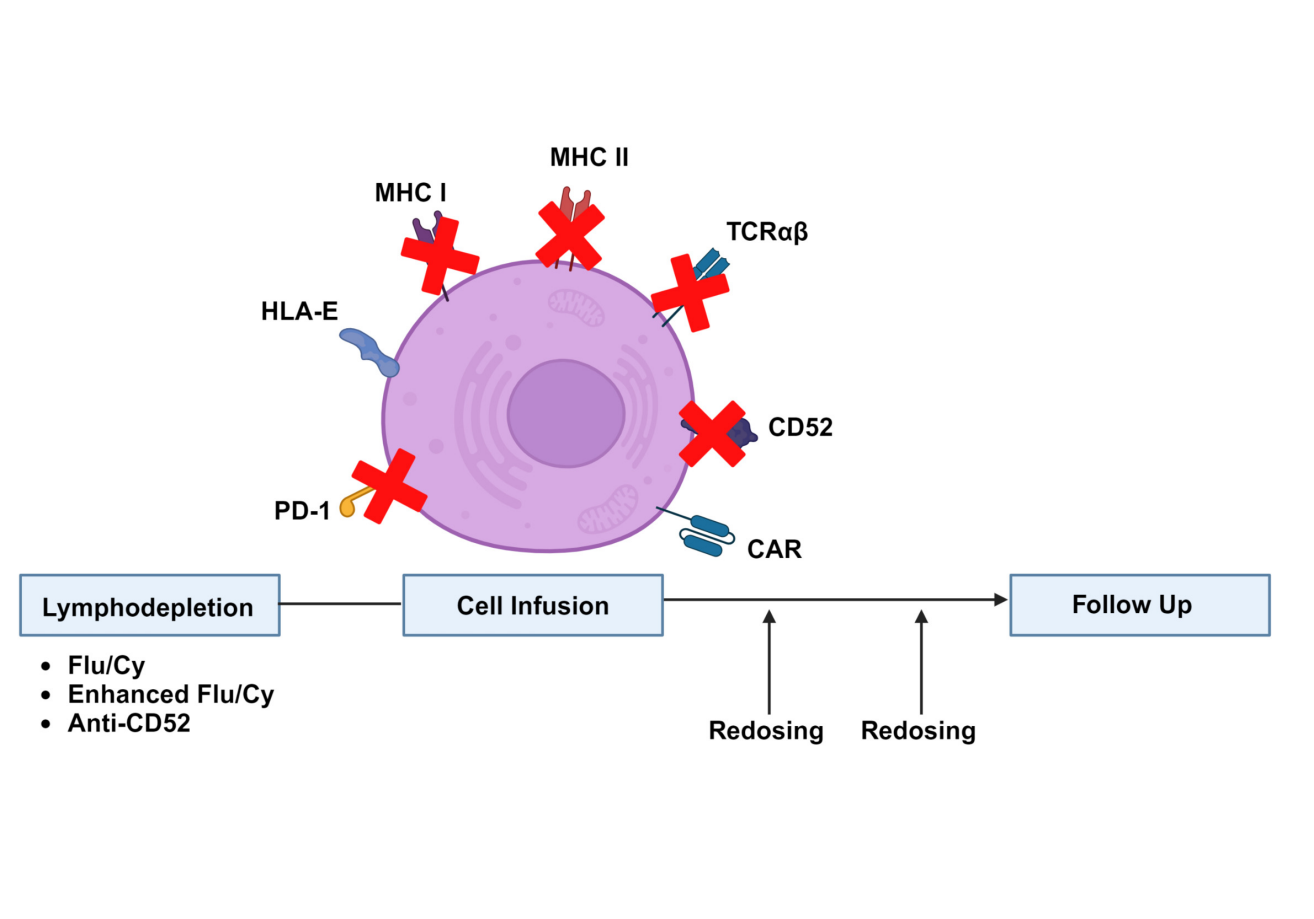
Strategies to improve allogeneic CAR T-cell expansion, persistence and efficacy. Flu: Fludarabine; Cy: Cyclophosphamide; MHC: Major histocompatibility complex’ HLA-E: Human leukocyte antigen E; PD-1: Programmed cell death protein 1

A significant challenge to clinical translation of allogeneic CAR T-cell therapies has been their limited expansion and persistence *in vivo*, particularly when compared to their autologous counterparts. CAR T-cell expansion following target antigen exposure is crucial to antitumor efficacy, and high peak CAR T-cell concentrations are associated with clinical responses in both autologous and allogeneic therapies.^16,41,42^ Pharmacokinetics of the currently available autologous products are strongly influenced by the nature of their co-stimulatory domains. Products with a 4-1BB co-stimulatory domain (e.g., tisagenlecleucel, lisocabtagene maraleucel) exhibit less robust expansion but durable persistence, while products with a CD28 co-stimulatory domain (e.g., axicabtagene ciloleucel, brexucabtagene autoleucel) exhibit rapid expansion but shorter persistence.^1^ Nevertheless, circulating CAR T-cells from autologous CD28-containing products can routinely be detected several months after treatment^16^ and CAR T-cells from a 4-1BB-containing product have even been detected 10 years after the initial infusion.^43^ In contrast, allogeneic CAR T-cell products with a 4-1BB co-stimulatory domain typically persist on the order of several weeks^35,41^ and there have been concerns regarding durability of response due to early relapses noted in some trials.^36,37^ Strategies to improve allogeneic CAR T-cell expansion and persistence include alterations to the lymphodepletion and dosing regimens, as well as genetic modifications intended to minimize rejection by the recipient immune system (**Figure 2**).

Prior to CAR T-cell infusion, a lymphodepleting conditioning regimen of fludarabine (Flu) and cyclophosphamide (Cy) is typically administered. This regimen is crucial for CAR T-cell expansion and functions through multiple mechanisms, including alterations in circulating cytokine levels and effects on recipient immune cells.^44–47^ Enhanced lymphodepletion consisting of higher doses of Flu and Cy (Flu 30 mg/m^2^/day x 4 days/Cy 1000 mg/m^2^/day x 3 days compared to standard Flu 30 mg/m^2^/day x 3 days/Cy 500 mg/m^2^/day x 3 days) resulted in a striking 80-fold improvement in CAR T-cell expansion.^37^ However, only 3/15 responses were ongoing at > 6 months duration, and infectious complications limited subsequent use of this lymphodepletion regimen. Another technique involves incorporating an anti-CD52 antibody into the conditioning regimen while knocking out CD52 in the allogeneic CAR T-cells to prevent these cells from being targeted. This strategy has been shown to improve CAR T-cell expansion and is under investigation in several ongoing clinical trials,^35,48,49^ although concerns regarding infectious complications remain. As enhanced lymphodepletion techniques result in deeper and longer suppression of recipient T-cell function compared to conventional lymphodepletion, they may also be associated with prolonged cytopenias and increased rates of atypical viral and fungal infections.^50^ Additional data regarding the type and frequency of these infections are needed to inform infection surveillance and antibiotic prophylaxis strategies for patients receiving enhanced lymphodepletion regimens. Of note, the possibility of repeat dosing using CAR T-cells generated from the same or a different donor presents exciting possibilities for consolidation of therapeutic responses and treatment of relapsed disease. At the present time, however, data regarding the optimal applications of this approach and associated lymphodepletion strategies are lacking.

Rejection by the recipient immune system has been implicated as a key factor limiting the expansion and persistence of allogeneic CAR T-cells.^18,51^ Although the precise mechanisms governing this process are not well understood, recipient T-cells, NK-cells and antibodies to the infused product are all thought to contribute. Several genetic modifications of allogeneic CAR T-cells have been proposed to minimize rejection by these components of the immune system. Recipient T-cells have been a primary focus of these efforts due to their well-established role in failure of hematopoietic stem cell transplants^52^ and the current impracticality of full human leukocyte antigen (HLA) matching of allogeneic CAR T-cell donors to recipients. Initial efforts to prevent T-cell mediated rejection included knockdown of beta-2 microglobulin to prevent major histocompatibility complex (MHC) class I expression^53,54^ and rejection by CD8^+^ T-cells. Despite strong preclinical rationale, this approach has not translated into dramatically improved clinical outcomes, with one recent trial reporting that 11/27 (41%) patients attained a complete response after receiving such a product, but only 5 of these patients maintained their response at 6 months.^55^ One possible explanation for these findings is that elimination of MHC I removes a key inhibitory signal for NK cells and renders the CAR T-cells more susceptible to NK cell-mediated rejection. To address this issue, several groups have proposed to upregulate the minimally polymorphic HLA-E molecule to inhibit NK cell activation while still preventing rejection by CD8^+^ T-cells.^56–58^ Other proposed strategies to minimize immune rejection include *CIITA* knockout to disrupt MHC class II expression necessary for CD4^+^ T-cell activation,^59,60^ and CD64 overexpression to prevent antibody-mediated cell destruction.^61^ Alternatively, expression of a CAR targeting antigens expressed on activated T-cells has been proposed as a means to improve CAR T-cell expansion through elimination of the recipient alloreactive T-cells responsible for CAR T-cell rejection.^62,63^ Allogeneic CAR T-cell products with combinations of these modifications have demonstrated promising persistence and efficacy in preclinical models^64,65^ and are currently being evaluated in clinical trials.

The techniques used to deliver and edit genes during the CAR T manufacturing process have also served as an important focus of research. While all the currently available CAR T-cell products use viral vectors to introduce the CAR transgene, there has been growing interest in virus-free delivery techniques, which would simplify the manufacturing process and overcome the limitations associated with viral gene delivery. These challenges include the limited size of viral gene inserts, difficulty in producing high titers of stable viral particles, and potential immunogenicity of virus-derived peptides.^66^ Transposon-based systems, such as Sleeping Beauty^67^ and piggyBac,^68^ have been successfully used to generate a variety of CAR T-cell products.^69–72^ These platforms involve the transfection (typically by electroporation) of T-cells with plasmids encoding the CAR of interest and a transposase which facilitates integration of CAR DNA into the genome. Transposon-based systems allow delivery of larger genes and are simpler to manufacture than viral vectors. However, they typically have lower transduction efficiencies and lack the ability to control the site of insertion into the genome. The importance of CAR insertion site was highlighted following the discovery of CAR T-cell-derived lymphomas in two patients receiving anti-CD19 products generated using the piggyBac transposon system in a phase 1 clinical trial.^73^ Genetic analysis showed a high transgene copy number in these lymphomas but no insertion into typical oncogenes. Although global changes in transcription predominantly correlated with gene copy number, the investigators did note transgene promoter-driven transcriptional upregulation of regions surrounding the insertion site, despite the use of insulator sequences surrounding the transgene. Other gene delivery strategies involving electroporation^74,75^ and self-assembled nanostructures^76,77^ also hold promise to simplify the CAR T-cell manufacturing process. However, the efficiency and persistence of the resulting genetic modifications remain important considerations when selecting a particular technique for CAR T-cell generation.

In addition to reliable gene delivery techniques, the number and complexity of genetic modifications involved in manufacturing allogeneic CAR T-cells requires the use of precise gene-editing technologies. Several allogeneic CAR T-cell products use transcription activator-like effector nucleases (TALENs), which are nucleases fused to sequence-specific DNA-binding domains that allow them to cut DNA at precise locations.^34,35,78^ Other products use gene editing platforms derived from the clustered regularly interspaced short palindromic repeats (CRISPR)-CRISPR associated protein 9 (Cas9) system, which uses a guide RNA strand to allow for selective editing of the genome.^55,79,80^ Although the details of the gene editing process are beyond the scope of this review, it is important to note that this aspect of manufacturing carries important regulatory and clinical implications. For instance, as gene editing technologies remain imperfect, and some modifications (e.g., *TRAC* knockout) are intended to minimize toxicity, there is a need to ensure that unedited or incompletely edited cells are not present in the final product. The optimal strategy for this purpose depends on the precise gene edits in question. However, FDA guidance suggests incorporating techniques that assess the intended functional outcomes of the genomic modifications.^81^ For products with TCR knockout, a purification step involving magnetic bead depletion of cells continuing to express the αβ TCR has proven effective at ameliorating the risk of GVHD.^33,82^ Additionally, there is concern that unexpected or off-target genomic alterations may result in development of a malignant clone. This concern led to a clinical hold of a trial involving ALLO-501A, an anti-CD19 allogeneic CAR T-cell product, after identification of a clonal chromosome 14 inversion in a patient who developed cytopenias following treatment.^83^ On further assessment, this rearrangement was not found to occur at the site of TALEN gene editing and, instead, appeared to be associated with the naturally occurring recombination-activating gene (RAG) enzyme implicated in physiologic V(D)J recombination. The clinical hold has since been lifted and ALLO-501A is currently being evaluated in a registrational phase 2 trial for treatment of relapsed/refractory large B-cell lymphoma (NCT04416984). This case highlights the challenges involved in clinical translation of gene editing technologies, and emphasizes the importance of detailed scientific analyses to elucidate the underlying causes of events noted in clinical trials.

## Alternative Cell Types

Although CAR T-cells have demonstrated impressive efficacy in hematologic malignancies, their toxicity profile and poor efficacy in solid tumors have prompted interest in other cell types whose physiological properties may be leveraged to overcome these challenges. Each cell type has unique characteristics which provide advantages in specific clinical applications (**Table 1**). The most extensively studied to date is the natural killer (NK) cell.^84,85^ Like T-cells, NK cells exhibit direct cytotoxicity through the release of lytic granules and activation of death receptors on the target cell.^86^ However, NK cells are not associated with development of GVHD, CRS or ICANS even in the absence of additional genetic modifications. Additionally, NK cells may be activated through CAR-independent mechanisms which could provide benefit with tumor types which do not homogeneously express a particular target antigen. Nevertheless, persistence of these cells and the durability of clinical responses remain a concern. Several different manufacturing strategies and genetic modifications have been proposed to optimize CAR NK-cell efficacy *in vivo*.

**Table 1.** Comparison of cell types used for engineered cellular therapies

**Cell Type**	**αβ T Cells**	**NK Cells**	**γδ T Cells**	**Macrophages**
**Source**	Peripheral blood UCB iPSCs	Peripheral blood UCB iPSCs	Peripheral blood UCB iPSCs	Peripheral blood UCB iPSCs
**Mechanism of Killing**	Exocytosis of lytic proteins Perforin Granzyme Release of pro-apoptotic receptor ligands Fas ligand TRAIL	Exocytosis of lytic proteins Perforin Granzyme Release of pro-apoptotic receptor ligands Fas ligand TRAIL	Exocytosis of lytic proteins Perforin Granzyme Release of pro-apoptotic receptor ligands Fas ligand TRAIL	Phagocytosis
**Modifications to Reduce GVHD Risk**	TCR Knockout	None	None	None
**Non-CAR Activation Mechanisms**	None (TCR knockout)	Activating/inhibitory surface receptors	Activating/inhibitory surface receptors	Activating/inhibitory surface receptors
**Toxicity Risk**	Higher	Lower	Lower	Lower

UCB: Umbilical cord blood; iPSCs: Induced pluripotent stem cells; TRAIL: TNF-related apoptosis-inducing ligand; TCR: T-cell receptor

As with T-cells, sources of NK cells for product manufacturing include peripheral blood mononuclear cells,^87^ umbilical cord blood^88^ and iPSCs.^89^ Appropriate cytokine stimulation is critical for proliferation of NK cells *in vitro* as well as antitumor efficacy and persistence *in vivo*. CAR NK-cells derived from umbilical cord blood and modified to constitutively express IL-15 have demonstrated antitumor activity in both animal models^88^ and clinical trials.^90^ Of note, no GVHD, CRS, ICANS or increase in the levels of inflammatory cytokines were noted during that study. Similar strategies, including the use of an IL-15 receptor fusion protein^91^ and exogenous IL-2 administration,^92^ have also been investigated in iPSC-derived CAR-NK cells. Stimulation with IL-12, IL-15 and IL-18 during NK cell expansion has been shown to induce a memory-like NK cell phenotype^93^ with functional benefits for antitumor activity.^87,94,95^ However, the application of this manufacturing process to the production of CAR NK-cells has not yet been studied. Although NK cells hold great promise as a platform for adoptive cell therapy, further studies are needed to identify the precise cellular modifications and clinical applications which will lead to optimal efficacy.

Other components of the innate immune system have also been proposed as cell therapy platforms due to their intrinsic cytotoxic properties. Unlike the αβ T-cells that predominate in current CAR T-cell products, γδ T-cells do not mediate GVHD and are capable of recognizing antigens in an MHC-unrestricted manner.^96,97^ A phase-1 study of anti-CD20 CAR γδ T-cells showed promising safety and efficacy, with 7/9 (78%) of patients achieving a complete response and no grade ≥3 CRS or ICANS noted.^98^ CAR macrophages have also been explored for use in solid tumors.^99,100^ These cells typically exert their cytotoxic effects through phagocytosis. However, they also produce a unique set of cytokines and chemokines which may be crucial to overcoming the immunosuppressive nature of the solid tumor microenvironment.^101^ Although development of these platforms is still in its early stages, they demonstrate the variety of cell types which may be harnessed to overcome the challenges associated with CAR T-cell therapy.

## Innovative Manufacturing Platforms

**Figure 3.**
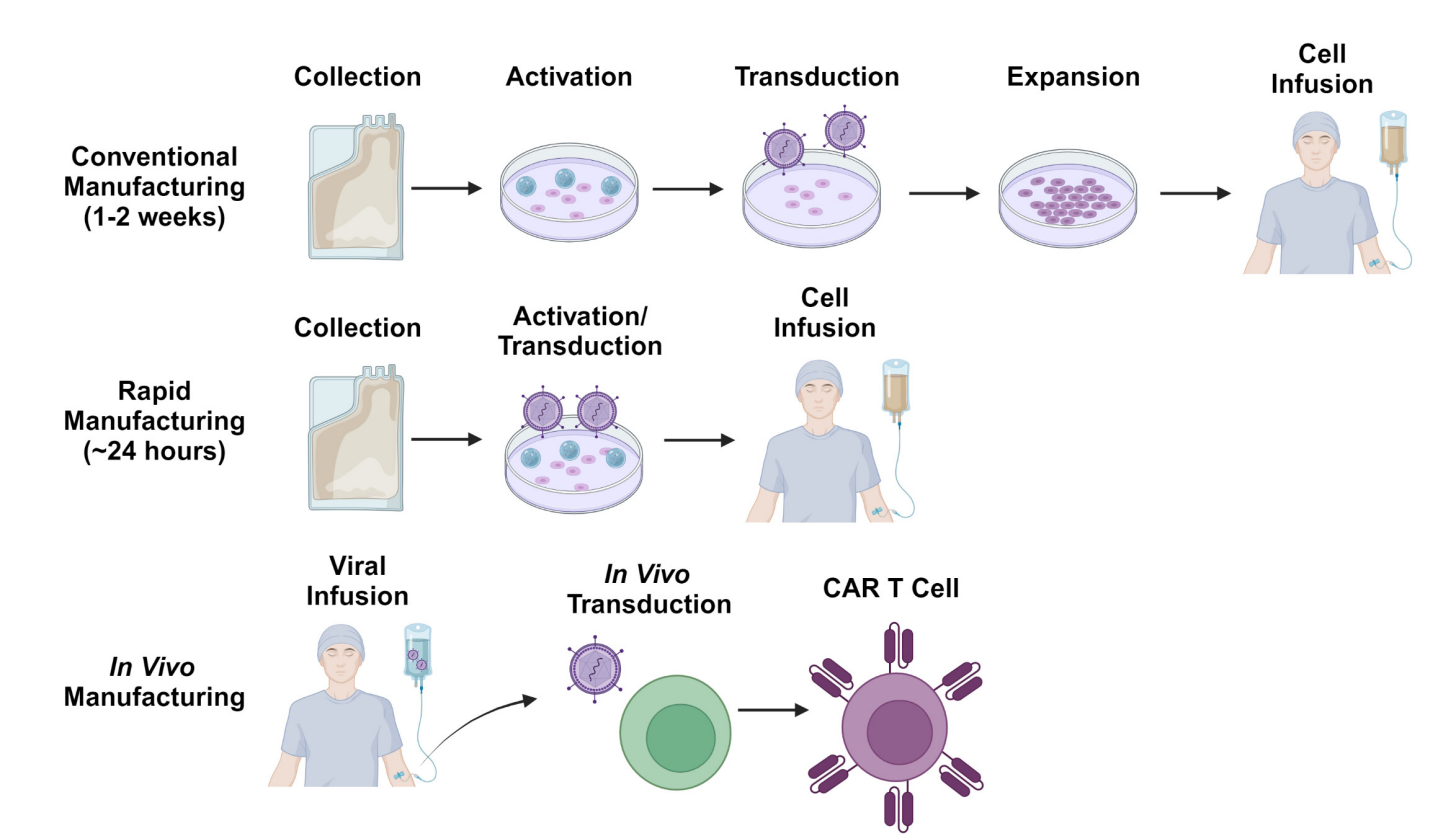
Innovative CAR T-cell manufacturing paradigms

Despite the potential advantages of allogeneic cell therapy, there remains considerable interest in improving autologous CAR T-cell manufacturing processes to make CAR T-cell production quicker and logistically simpler (**Figure 3**). One such strategy involves de-centralizing the manufacturing process and allowing each treatment center to generate their own CAR T-cells on-site. As most treatment centers will not have access to good manufacturing practices (GMP)-compliant production facilities, several automated tabletop GMP-compliant manufacturing systems have been implemented for this purpose.^102^ This paradigm has demonstrated feasibility in the clinical trial setting^103^ and multicenter trials using these manufacturing platforms are ongoing. Nevertheless, the time required for T-cell activation, transduction and expansion, as well as the extensive testing required by regulatory authorities before release of each dose remain significant challenges.

The currently available CAR T-cell products utilize manufacturing processes which typically take 7-14 days. However, there has been growing interest in manufacturing platforms which can generate CAR T-cells more rapidly, within 24 hours in some cases.^104^ One such approach involves the transduction of non-activated T-cells along with other modifications to compensate for the poor transduction efficiency typically associated with non-dividing cells.^105^ Cells manufactured using a similar process have more desirable stem-like phenotypes and have shown promising efficacy in clinical trials.^106–108^ Other innovative platforms include the generation of CAR T-cells on implantable tissue scaffolds^109^ and *in vivo* generation of CAR T-cells through the use of viral or nanoparticle-based gene delivery systems.^110–113^ While still in early stages of development, these strategies exemplify some of the innovative solutions proposed to overcome the logistical barriers to CAR T-cell therapy.

## Conclusions

Despite its demonstrated efficacy, the impact of autologous CAR T-cell therapy has been limited by cost, toxicity and logistical challenges. Innovative cell therapy platforms, including the use of allogeneic donors, alternative cell types, and rapid manufacturing protocols, hold promise to overcome these limitations. Each of these strategies has unique advantages and disadvantages which will determine their ultimate role in clinical practice. As the barriers to CAR T-cell therapy are overcome and novel targets are discovered, this form of treatment is sure to play an increasing role in the management not only of hematologic malignancies, but of solid tumors and non-malignant disorders as well. This broad range of potential applications highlights the exciting possibilities and immense promise for the future of engineered cell therapies.

### Authors’ Contribution per CRediT

Conceptualization: Andrew P Jallouk (Equal), Salyka Sengsayadeth (Equal), Bipin N Savani (Equal), Bhagirathbhai Dholaria (Equal), Olalekan Oluwole (Equal). Methodology: Andrew P Jallouk (Equal), Salyka Sengsayadeth (Equal), Bipin N Savani (Equal), Bhagirathbhai Dholaria (Equal), Olalekan Oluwole (Equal). Writing – original draft: Andrew P Jallouk (Lead), Olalekan Oluwole (Supporting). Writing – review & editing: Andrew P Jallouk (Equal), Salyka Sengsayadeth (Equal), Bipin N Savani (Equal), Bhagirathbhai Dholaria (Equal), Olalekan Oluwole (Equal).
